# Comparison of Two Anesthetic Methods for Intravitreal Ozurdex Injection

**DOI:** 10.1155/2015/861535

**Published:** 2015-04-09

**Authors:** V. Levent Karabaş, Berna Özkan, Çiğdem Akdağ Koçer, Özgül Altıntaş, Dilara Pirhan, Nurşen Yüksel

**Affiliations:** Ophthalmology Department, Faculty of Medicine, Kocaeli University, 34885 Kocaeli, Turkey

## Abstract

*Purpose*. To determine whether subconjunctival lidocaine injection maintains additional anesthetic effect during intravitreal Ozurdex injection. *Methods*. 63 patients who were diagnosed as central or branch retinal vein occlusion and planned to receive Ozurdex injection for macular edema were prospectively included in the study. The patients were randomized into one of the two anesthetic groups. The first group received topical proparacaine drop and lidocaine applied pledget. The second group received subconjunctival lidocaine injection in addition to the anesthetics in group 1. *Results*. Mean pain score was 1.90 ± 2.39 in group 1 and 1.71 ± 2.09 in group 2 (*p* = 0.746). Mean subconjunctival hemorrhage grade was 1.67 ± 0.17 in group 1 and 0.90 ± 0.14 in group 2 (*p* = 0.001). There was no relationship between the amount of subconjunctival hemorrhage and pain score of the patients. *Conclusions*. There was no difference in pain scores between the two anesthetic methods. The addition of subconjunctival lidocaine injection offered no advantage in pain relief compared to lidocaine-applied pledgets.

## 1. Introduction

Intravitreal injection has become an important treatment choice in many vision-threatening diseases. Various pharmacologic agents such as antibiotics, steroids, and antifungal and antiviral drugs can be delivered into the vitreous cavity by this method [1]. Recently, antivascular endothelial growth factor (VEGF) drugs gained popularity especially in treating age-related macular degeneration and diabetic macular edema [2]. Thus intravitreal injections have become one of the most common medical procedures.

Intravitreal injections are usually performed as an office procedure, and topical and/or local anesthesia is used during the injection. The most common anesthesia techniques are topical anesthetic eye drops, anesthetic-applied pledget placed on the conjunctiva, and subconjunctival anesthetic injection [3]. Although all of these techniques are found to be effective in the previous studies, there is no consensus for the most effective method [4–7]. On the other hand the studies that investigated the effect of anesthesia method in intravitreal injections usually evaluated the small-sized needles. For this reason, the effect of anesthesia in intravitreal Ozurdex injection is not known.

Ozurdex (Allergan, Inc., Irvine, CA) is an intravitreal implant containing 700 *μ*g of dexamethasone in a slow-release preparation. Key features of the drug delivery system are the sustained-release formulation of the polylactic acid-coglycolic acid (PLGA) matrix material, which dissolves completely in vivo, and the single-use applicator for intravitreal placement. The United States Food and Drug Administration (FDA) and European Union (EU) approve it for the intravitreal treatment of macular edema. It is indicated for the treatment of adult patients with macula edema following retinal vein occlusion (RVO) (either branch or central) and inflammation of the posterior segment of the eye presenting as noninfectious uveitis. Furthermore, clinical efficacy has been documented in other diseases, such as diabetic macular edema (DME) and persistent macular edema (ME) associated with uveitis or Irvine-Gass syndrome [8].

The indications for intravitreal Ozurdex implantation have broadened in the recent years. Consequently, increasing numbers of Ozurdex implantation are administered. During implantation procedure, patient comfort and eye stabilization are very important to avoid complications related to application; calm and painless patient can allow us to complete Ozurdex implantation without any problem. Some of the surgeons are using subconjunctival anesthesia to reduce the pain that occurs while Ozurdex applicator is penetrating through the sclera on pars plana. It is arguable if subconjunctival anesthesia really reduces pain or causes more conjunctival hemorrhage and edema that could prevent finding the proper injection site and control the leakage after injection.

We conducted a prospective randomized clinical trial comparing the patients' comfort and pain with or without subconjunctival anesthesia during intravitreal Ozurdex implantation procedure.

## 2. Materials and Methods

This prospective randomized study included 63 patients who were diagnosed as central or branch retinal vein occlusion. The patients underwent intravitreal Ozurdex injection for their macular edema related to their diagnosis. All patients were naïve to any kind of intravitreal injection before enrollment. The Ethical Committee of Kocaeli University Medical Faculty approved this study. After the patients provided a written consent, each patient was randomized into one of the two anesthetic groups. The first group received topical proparacaine drop and 4% lidocaine-applied pledget. The second group received subconjunctival lidocaine injection in addition to the anesthetics in group 1.

### 2.1. The Anesthesia Methods and Injection

First, topical proparacaine hydrochloride ophthalmic solution was instilled in eyes of the patients in both groups. Then proparacaine-applied sterile pledgets were placed into upper and lower conjunctival sac of the patients in both groups for 1 minute. Povidone-iodine was used to prepare the ocular surface for preinjection antisepsis and the periocular area was covered with an ophthalmic drape. A lid speculum was placed. All eyes were irrigated with 10% povidone-iodine after draping. A second irrigation with sterile 0.9% NaCl was performed. The patients in group 1 (31 patients) received subconjunctival lidocaine injection before Ozurdex implantation, and the patients in group 2 (32 patients) did not receive any anesthesia other than topical proparacaine hydrochloride and 4% lidocaine-applied pledget.

This step was followed by the insertion of the Ozurdex implant into the vitreous cavity through the pars plana using a customized, single-use 22-gauge applicator in both groups by the same surgeon. Standard eye calipers were used to measure the distance from the limbus (3.5 mm for the pseudophakic patients, 4 mm for the phakic patients). Injections were performed in the superotemporal quadrant of the each eye.

### 2.2. Hemorrhage and Pain Assessment

After the subconjunctival lidocaine application the surgeon assessed the grade of the subconjunctival hemorrhage using a scoring system (none = 0, minor = 1, moderate = 2, and severe = 3).

Approximately 15 minutes after the injection, a masked ophthalmology resident administered a standard questionnaire to evaluate the patients' pain level during the injection. We used a Visual Analog Pain score survey [9], where 0 = no pain/no distress and 10 = agonizing pain/unbearable distress. The VAS scale is the methodology that is most commonly used for the evaluation of pain severity and relief and has been employed in similar studies measuring ocular comfort [10, 11].

### 2.3. Statistical Analysis

All analyses were performed using SPSS version 16.0. Independent sample *t*-test was used in the statistical analysis of the two groups. The paired sample *t*-test was used in intragroup comparisons. Pearson's correlation test was used in assessment of the correlation between the subconjunctival hemorrhage grade and the pain scores of the patients. *p* values those were smaller than 0.05 were considered as statistically significant.

## 3. Results

There was no statistically significant difference between the groups in age and gender. The mean age was 63.1 ± 2.1 in group 1 and 60.6 ± 2.7 in group 2 (*p* = 0.337). There were 16 male and 16 female in group 1 and 16 male and 15 female patient in group 2 (*p* = 0.827).

Mean pain score comparison between the patients with or without subconjunctival anesthesia showed no statistically significant difference. Mean pain score was 1.90 ± 2.39 in group 1 and 1.71 ± 2.09 in group 2 (*p* = 0.746).  Table 1 shows the number of patients in each pain score.

Subconjunctival hemorrhage was higher in subconjunctival lidocaine group (group 1). Mean subconjunctival hemorrhage score in group 1 was 1.67 ± 0.17 and 0.90 ± 0.14 in group 2. The difference between the groups was statistically significant (*p* = 0.001).  Table 2 shows the number of patients in each subconjunctival hemorrhage grade.

There was no relationship between the amount of subconjunctival hemorrhage and pain score of the patients. Pearson's correlation test did not reveal any statistically significant correlation (*p* = 0.066 and *r* = 0.233).

## 4. Discussion

The use of corticosteroids for the treatment of ocular inflammatory diseases was first described in the early 1950s. Corticosteroids have anti-inflammatory, antiangiogenic, and antipermeability properties that make them an important therapeutic option for a variety of posterior segment diseases [12]. Based on experimental studies, clinical observations, and pathogenic considerations, the intravitreal delivery of steroids has been suggested to locally suppress intraocular inflammation, proliferation of cells, and neovascularization [13]. Intravitreal delivery of corticosteroids has allowed many posterior segment diseases to be locally treated without the adverse systemic side effects. An increasing number of ophthalmologists use intravitreal steroids for the treatment of various posterior segment disorders, especially when traditional therapeutic methods have failed.

A variety of methods have been proposed that achieve longer duration of pharmacologic effect with lower administration frequency and minimal side effects of the intravitreal steroids. Novel agents including preservative-free and sustained-release intravitreal implants such as Ozurdex are currently approved for ocular use and are being further evaluated for the treatment of RVO, DME, uveitis, and AMD. Due to a potential for greater potency, dexamethasone is being evaluated alone or in combination with anti-VEGFs as promising options in the emerging armamentarium for the treatment of several retinal diseases [8]. The safety and tolerability of a sustained-release implant are particularly important due to the long duration of exposure to the drug and the drug vehicle.

The intravitreal injections are usually administered as a part of the office procedure. In order to maintain a successful injection the patient should be comfortable and relaxed. This can be achieved by a minimally painful injection. The American Society of Retina Specialists Preferences and Trends Survey from 2010 revealed that 25.44% of the retina specialists use topical anesthetic drop, 25.15% use topical viscous anesthetic, 26.33% use topical anesthetic (drop or viscous) + pledget, and 22.19% use subconjunctival injection of anesthetic for intravitreal injections [3]. Canadian Ophthalmological Society carried out a survey for retina specialists in Canada and evaluated the intravitreal injection techniques. They found that 90% of retina specialists routinely used topical proparacaine, lidocaine, or tetracaine drops in preparation for intravitreal injection. Twenty-five percent routinely used topical lidocaine gel, and 16% used it infrequently. Twenty-three percent routinely used a pledget soaked with tetracaine or proparacaine, and 28% used pledgets infrequently. Twenty-three percent routinely used subconjunctival lidocaine injection, and 43% used this technique infrequently [14].

Recently, few studies assessed patient comfort using different kinds of anesthetic methods during intravitreal injections. In most of these studies it was found that all of these methods were effective [4–7]. Most of these studies have shown no significant difference in pain score between pledget, subconjunctival injection, or topical drops [5, 6, 15–17]. Blaha et al. compared the effectiveness of proparacaine, tetracaine, lidocaine pledget, and subconjunctival injection of lidocaine. They found no statistical difference in injection or total procedure pain scores between these methods [5]. Davis et al. also evaluated the difference in anesthetic effect between topical proparacaine drops, 4% lidocaine-applied cotton tipped swabs, or 3.5% lidocaine gel. After the injection they asked the patients to grade the discomfort associated with 3 components of the injection procedure: (1) the lid speculum; (2) the needle insertion; and (3) the burning sensation from the 5% povidone-iodine solution. They did not find any difference between the groups in any of the factors that might cause discomfort during the injection [15]. However, there are also a few studies claiming that there was difference between the anesthesia methods. LaHood et al. compared the anesthetic effectiveness of topical gel, subconjunctival and combination of topical gel, and subconjunctival anesthesia for intravitreal injection in 120 consecutive patients. Their results showed that the group receiving topical gel anesthetic produced significantly higher pain scores compared to both of the other groups [18]. Blaha et al. did not find statistically significant difference between topical proparacaine drops, pledget of 4% lidocaine, and subconjunctival injection of 2% lidocaine. But they reported that proparacaine drops had the lowest average combined with pain score [19].

In most of these studies, researchers evaluated the anesthetic methods in intravitreal injection of ranibizumab (Lucentis, Genentech), bevacizumab (Avastin, Genentech), or triamcinolone (Kenalog, Bristol-Myers Squibb). In order to perform these injections a smaller sized needle was used (27.5–32 gauges). However, Ozurdex injection is different than other intravitreal injections. It has a larger needle, which is 22 gauges. While inserting the Ozurdex needle it feels blunter. The long axis of the applicator should be held parallel to the limbus, and the sclera should be engaged at an oblique angle with the bevel of the needle up (away from the sclera) to create a shelved scleral path. The tip of the needle is advanced within the sclera for about 1 mm (parallel to the limbus) and then redirected toward the center of the eye and advanced until penetration of the sclera is completed and the vitreous cavity is entered (the package insert is available online at http://www.allergan.com/assets/pdf/ozurdex_pi.pdf). This procedure is more complicated than the quick fine-gauge “pinprick” of other intravitreal therapies. This might result in higher pain perception than the other intravitreal injections. For this reason, patients might distinguish the difference between the anesthetic techniques in this higher pain level. In our study we compared the anesthetic methods in intravitreal Ozurdex injection. We found that subconjunctival lidocaine injection did not maintain additional decrease in pain.

Kozak et al. compared the gel anesthesia using different needle lumens. They observed no difference between 27.5-gauge and 30-gauge needles, but these needles are also smaller than Ozurdex applicator [7]. For this reason, they cannot be compared with Ozurdex injection. While we were planning our study, we thought that topical proparacaine drops might not be enough as a single anesthetic method in Ozurdex implantation because Ozurdex has a larger applicator. We combined proparacaine drops with lidocaine-applied pledgets in all patients and an additional subconjunctival lidocaine injection in another group of patients. The advantage of using the pledgets is the application of high concentration of anesthetic to the site of injection. Pledgets might also have some disadvantages, such as irritation, corneal edema, and corneal epithelial defects. None of the patients in our study complained about the irritation, and we did not observe any corneal edema and epithelial defects. In order to avoid these problems smaller pledgets might be used. However smaller pledgets could be lost in the fornix, and to go fishing around the fornix to retrieve a pledget might cause additional discomfort for the patient. We believe that comparison between single use of topical proparacaine and 4% lidocaine-applied pledgets in Ozurdex injection must be studied in further studies.

In our study we found higher incidence of subconjunctival hemorrhage in patients that underwent subconjunctival injection. It might be a minor complication; however patients find the appearance of their eyes bothersome. Additionally, it might cause difficulty in injection especially when it is combined with the chemosis. It makes marking the injection site location more difficult [13]. Other complications that were reported to be related to subconjunctival lidocaine are inadvertent intravitreal injection and hypersensitivity [20, 21]. Subconjunctival hemorrhage and chemosis might also lead to inadvertent intravitreal injection. We did not observe any of these complications. However we believe that taking the risks that might be caused by subconjunctival chemosis and hemorrhage is unnecessary because subconjunctival lidocaine injection does not maintain additional pain reduction. There is also a controversy about the effect of subconjunctival anesthesia on endophthalmitis rate. Tustin et al. claim that subconjunctival lidocaine is bactericidal and maintains a clinically important antiseptic effect. As a result, they suggest that application of subconjunctival lidocaine may reduce the incidence of endophthalmitis after intravitreal injection [22]. On the other hand in another prospective randomized case control study, subconjunctival anesthesia is found to be a potential risk factor for postinjection endophthalmitis. The authors hypothesize that compromising the conjunctival surface before injection allows the introduction of bacteria into the subconjunctival space. This could act as a source of infection for postinjection endophthalmitis [23]. We should also consider this risk while performing subconjunctival lidocaine anesthesia.

In conclusion, there are different methods for pain reduction during intravitreal injections. However, there has been no method repeatedly shown to be superior in controlling pain. To the best of our knowledge, this study is the first study that compares the anesthesia techniques in intravitreal Ozurdex implantation. Our study showed that subconjunctival lidocaine injection is not mandatory in reducing pain during Ozurdex implantation. On the contrary, it causes conjunctival bleeding and chemosis in the injection site, which might cause difficulty in injection. We believe that future investigations to compare the local anesthesia techniques should be carried out.

## Figures and Tables

**Table 1:** The patients' pain level was evaluated by using a Visual Analog Pain score survey, where 0 = no pain/no distress and 10 = agonizing pain/unbearable distress. The number of patients in each pain score level is shown below. There was no statistically significant difference in pain score among the groups (*p* = 0.746).

	Group 1	Group 2
Pain score 0	9	10
Pain score 1	12	10
Pain score 2	3	6
Pain score 3	3	4
Pain score 4	1	2
Pain score 5	—	—
Pain score 6	—	1
Pain score 7	2	—
Pain score 8	—	—
Pain score 9	2	—
Pain score 10	—	1

**Table 2:** The number of patients in each subconjunctival hemorrhage grade in both groups is demonstrated. The patients that received additional subconjunctival anesthesia (group 1) presented with higher levels of subconjunctival hemorrhage compared to the patients that received only topical and pledged anesthesia (*p* = 0.001).

Subconjunctival hemorrhage	Group 1	Group 2
Grade 0	3	10
Grade 1	12	16
Grade 2	10	3
Grade 3	6	2
Grade 4	1	—
